# The effect of target and non-target similarity on neural classification performance: a boost from confidence

**DOI:** 10.3389/fnins.2015.00270

**Published:** 2015-08-05

**Authors:** Amar R. Marathe, Anthony J. Ries, Vernon J. Lawhern, Brent J. Lance, Jonathan Touryan, Kaleb McDowell, Hubert Cecotti

**Affiliations:** ^1^Translational Neuroscience Branch, US Army Research Laboratory, Human Research and Engineering DirectorateAberdeen Proving Grounds, MD, USA; ^2^Department of Computer Science, University of Texas at San AntonioSan Antonio, TX, USA; ^3^Intelligent Systems Research Centre, School of Computing and Intelligent Systems, University of UlsterLondonderry, UK

**Keywords:** confidence, EEG, classification, single-trial analysis, rapid serial visual presentation, brain-computer interface

## Abstract

Brain computer interaction (BCI) technologies have proven effective in utilizing single-trial classification algorithms to detect target images in rapid serial visualization presentation tasks. While many factors contribute to the accuracy of these algorithms, a critical aspect that is often overlooked concerns the feature similarity between target and non-target images. In most real-world environments there are likely to be many shared features between targets and non-targets resulting in similar neural activity between the two classes. It is unknown how current neural-based target classification algorithms perform when qualitatively similar target and non-target images are presented. This study address this question by comparing behavioral and neural classification performance across two conditions: first, when targets were the only infrequent stimulus presented amongst frequent background distracters; and second when targets were presented together with infrequent non-targets containing similar visual features to the targets. The resulting findings show that behavior is slower and less accurate when targets are presented together with similar non-targets; moreover, single-trial classification yielded high levels of misclassification when infrequent non-targets are included. Furthermore, we present an approach to mitigate the image misclassification. We use confidence measures to assess the quality of single-trial classification, and demonstrate that a system in which low confidence trials are reclassified through a secondary process can result in improved performance.

## Introduction

The application space for brain computer interaction (BCI) technologies is rapidly expanding with improvements in technology. For example, the use of BCI systems for image triage have enabled image analysts to detect targets in large aerial photographs faster and more accurately than traditional standard searches (Gerson et al., 2006; Parra et al., 2008; Sajda et al., 2010; Pohlmeyer et al., 2011; Zander and Kothe, 2011). Systems that incorporate neural activity to enhance visual target identification often utilize a rapid serial visual presentation (RSVP) paradigm in which analysts are shown a sequence of images in rapid succession (e.g., 2–10 Hz)(Potter, 1976; Chun and Potter, 1995). The analyst's task is to detect predefined targets occurring with low frequency in a series of frequent background (i.e., distractor) stimuli. When a target is detected in an image, a tell-tale neural response commonly associated with the P300 event-related potential (ERP) is evoked and classified by the BCI system (Pohlmeyer et al., 2011). Each image in an RSVP task is classified based on the neural response of the analyst and those that are deemed most likely to contain targets are triaged for subsequent interrogation by the analyst. By using the RSVP paradigm, it is possible for an analyst to quickly sort through many images.

Previous studies using RSVP tasks for rapid target detection have primarily focused on the two-class discrimination problem of detecting target images within a set of distractor images (Gerson et al., 2006; Bigdely-Shamlo et al., 2008; Parra et al., 2008; Sajda et al., 2010; Touryan et al., 2010, 2011; Cecotti et al., 2011; Yu et al., 2011, 2012; Marathe et al., 2013, 2014b). However, in many real-world environments there are likely to be a subset of distractor stimuli that share physical and semantic features with the target stimuli (e.g., consider a non-target elk vs. a target deer in a dense ensemble of forest imagery). While ERP studies have analyzed the neural features evoked by rare non-targets within a series of rare targets and frequent background distractors using simple classes of stimuli (e.g., letters and colored shapes) (Polich and Comerchero, 2003), it is unknown if similar effects occur in complex imagery more similar to real-world settings. Moreover, little research has been done to evaluate how current neural-based classification algorithms perform when two infrequent classes of stimuli with the same features (i.e., target and non-target) are presented in a sequence of frequently occurring distractor images. It is possible that many classification algorithms used for RSVP target detection studies are sensitive to neural features primarily associated with the detection of infrequent stimuli rather than target detection/recognition, resulting in drastically reduced performance.

The RSVP-based image triage process uses a measure of confidence in the classifier through the probability score as a means of quantifying the certainty of a decision. That is, the probability that a particular image is a target provides information regarding the likelihood a target was presented. The importance of confidence in systems with low signal-to-noise properties has long been understood in decision theory (Bernoulli, 1954; Pascal and Krailsheimer, 1968; Lehmann, 2012) and control communities (Olson et al., 2013; Tsiligkaridis et al., 2014) and peripherally exists in current instantiations of image triage BCIs (Gerson et al., 2006; Huang et al., 2008; Mathan et al., 2008; Sajda et al., 2010). Additional uses of confidence measures in BCIs are demonstrated through the rejection of particular trials from analysis or the use of algorithms for the removal of artifacts. Thus, while the use of confidence measures for target-detection BCIs is not new, previous studies have not explicitly described their methods for deriving the confidence metric, and have not quantified the accuracy of their confidence estimates or the unique contribution of confidence itself.

This study explores how current RSVP-based BCI technologies may function in more complex task environments by adding infrequent non-target images that are not task relevant, but which are physically and semantically similar to targets to presentations with rare targets and frequent background distractors. In the first half of the paper, we examine participants' ability to detect targets under two conditions: first when targets are the only infrequent image class presented and second, when the targets are presented with infrequent non-targets in a standard RSVP task. Our analysis encompasses behavior, averaged ERPs, and single-trial classification of EEG data. The results demonstrate that both behavioral and single-trial classification performance of target images decline with the introduction of rare visually-similar non-target stimuli. We also examine the effects of using trial-by-trial confidence measures derived from the relationship between individual classifier outputs and the discriminating threshold between targets and non-targets to mitigate the drop in classifier performance. These results provide a unique perspective into how methods for EEG classification of visual imagery may perform in more complex scenarios and the importance of incorporating confidence.

## Methods

### Participants

Eighteen participants volunteered for the current study. Participants reported normal or corrected-to-normal vision and no history of neurological problems. Due to excessive artifacts in the EEG data, one participant was excluded from analysis. The resulting 17 participants had an average age 34.9 years, 14 were male, and all participants were right handed with the exception of one left handed male.

The voluntary, fully informed, written consent of the persons used in this research was obtained as required by federal and Army regulations (U.S. Department of the Army, 1990; U.S. Department of Defense Office of the Secretary of Defense, 1999). The investigator has adhered to Army policies for the protection of human subjects (U.S. Department of the Army, 1990). All human subjects testing was approved by the Institutional Review Board of the United States Army Research Laboratory.

### Stimuli and procedure

**Figure 1:**
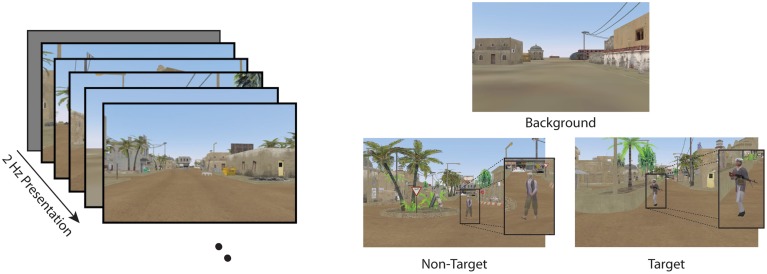
**RSVP task and stimuli in the current experiment**. Participants were required to detect target images while ignoring non-targets and background distractors.

Participants were seated 75 cm from a monitor and viewed a series of images from a simulated desert metropolitan environment in a RSVP paradigm (Figure 1). Images (960 × 600 pixels, 96 dpi, subtending 36.3° × 22.5°) were presented using E-prime software for 500 ms (2 Hz) with no inter-stimulus interval.

Data were analyzed from two conditions for all participants: Target Only (TO) and Target and Non-Target (TN). The TO condition contained only background distractors (background scenes of a dessert metropolitan environment) and target images (background scenes with a person carrying a weapon). The TN condition contained non-target distractor stimuli (background scene with a person without a weapon) along with both background and target stimuli. (See Figure 1 for examples of the stimuli). Target stimuli (both TN and TO conditions) and non-target distractor stimuli (TN condition only) were never presented back to back. At least two background stimuli were required to follow any target or non-target stimulus to avoid issues with the attentional blink (Raymond et al., 1992; Chun and Potter, 1995). In both the TO and TN conditions, participants were instructed to press a button on a serial response box as rapidly and accurately as possible with their dominant index finger when they detected a target. Participants were also instructed to silently count the number of targets they detected and report this number at the end of each block.

Each condition contained six blocks of RSVP image sequences. Each block was a 2-min image sequence in the TO condition and a 2 min and 14 s sequence in the TN condition. The inter-block rest period was self-paced after a mandatory 10 s pause to report the target count. Each block began with a visual 5-s count-down presented at the center of the display. Participants were told to fixate toward the center of the display as all target and non-target stimuli appeared within 6.5° of the image center and would not appear on top of or occluded by buildings and trees or in windows. Block order was counterbalanced across participants. The individual blocks served to break up the RSVP presentation and allow subjects to periodically rest. Thus, data from the six blocks within each condition were concatenated and analyzed as a whole.

The target to distractor ratio was 1:20 in the TO condition and 1:14 in the TN condition. The non-target to distractor ratio in the TN condition was also 1:14. Participants were not aware of stimuli contingencies. Participants were given one block of practice on each RSVP stimulus condition and were required to correctly report at least 75% of targets to begin the experiment. All participants needed only one practice block in each condition to satisfy this requirement.

### EEG recording and preprocessing

Electrophysiological recordings were digitally sampled at 1024 Hz from 64 scalp electrodes arranged in a 10-10 montage using a BioSemi Active Two system (Amsterdam, Netherlands). Impedances were kept below 25 kΩ. External leads were placed on the outer canthus of each eye and above and below the right orbital fossa to record EOG. Continuous EEG data were pre-processed using EEGLAB (Delorme and Makeig, 2004). The EEG data were referenced to the average of the left and right earlobes, decimated to 512 Hz, and digitally filtered 0.1–50 Hz.

Gross artifacts were removed through visual inspection of the continuous EEG data. Sections marked as artifacts were excised from the data. Subsequently, independent component analysis (ICA), (Jung et al., 2000) was run. Independent components related to eye movements or muscle activity were manually identified and removed. The time series data resulting from the ICA-based cleaning was used for all further analyses.

For single-trial classification, the signal was first bandpass filtered (Butterworth filter of order 4) with cutoff frequencies at 1 and 10.66 Hz and then downsampled to 32 Hz. This new sampling rate was chosen based on the sampling frequency used by the winning team of the competition in the 2010 IEEE Workshop on Machine Learning for Signal Processing (MLSP) (Leiva and Martens, 2010).

### Behavioral analysis

To quantify the behavioral performance, any button press that occurred between 200 and 1000 ms after a target or non-target stimulus was attributed to that trial. Button presses attributed to target trials were counted as hits, and all others as false positives. Reaction times were calculated as the time between stimulus presentation and button press.

Hits (Hit), misses (Miss), correct rejects (CorrectReject), and false positives (FP) were calculated for each subject. The correct rejects and false alarms were calculated separately for non-targets and distractor trials in order to investigate the effect of adding the non-target stimuli to the behavioral performance. These values were used to calculate d′ (d-prime), an index of accuracy that accounts for response bias (Green and Swets, 1966), for each subject:

(1) $$\begin{array}{l} HR=\;\frac{Hit}{Hit+Miss}\;FPR=\;\frac{FP}{FP+CorrectReject} \end{array}$$

(2) $$\begin{array}{l} d^{\prime }=\;Z\left(HR\right)-\;Z\left(FPR\right) \end{array}$$

Where the function *Z*(*p*), *p* ∈ [0,1], is the inverse of the cumulative Gaussian distribution.

### ERP analysis

ERP data were processed and analyzed using ERPLAB (Lopez-Calderon and Luck, 2014). Artifact free data were epoched [–500, 1000] ms around stimulus onset and binned according to the experimental condition. ERPs were baseline corrected by subtracting the mean of the activity of each channel from [–500, 0] ms from the epoched data. Only hits and correct rejections were included in the ERP analysis. ERPs were calculated for each stimulus type (background distractors, targets, non-targets). P3 amplitude (400–800 ms) was separately calculated for each subject in each experimental condition at electrode Pz. The time segment analyzed was chosen based on the grand average target ERP waveforms, which showed the maximum P3 amplitude occurring over electrode Pz 400–800 ms post-stimulus.

### Single-trial classification

In order to quantify the effects of adding rare, target-like non-target stimuli at the single-trial level, EEG data were epoched to [0, 1000] ms, timelocked to stimulus onset, spatial filtered using xDAWN (Rivet et al., 2009), and classified with Bayesian linear discriminant analysis (Hoffmann et al., 2008) [collectively referred to as XD+BLDA (Rivet et al., 2009; Cecotti et al., 2011, 2012, 2015)].

#### XD+BLDA

The xDAWN algorithm is a spatial filtering algorithm that identifies a linear combination of the raw neural signals that maximizes the signal to noise ratio between targets and non-targets. Let U ∈ R${}^{N_{s}\times N_{f}}$ be the spatial filters, where *N*_*s*_ is the total number of sensors and *N*_*f*_ is the number of spatial filters. The signal after spatial filtering is defined by *X*_*filt*_ = *XU* where $\text{X}\in {\text{R}}^{N_{t}\times N_{s}}$ is the recorded signal, *N*_*t*_ is the number of sampling points. The expected waveform is considered spatially stable over time for the spatial dimension reduction step.

In this framework, an algebraic model of the enhanced signals *XU* is composed of three terms: the ERPs evoked by the targets (*D*_1_*A*_1_), a response common to all stimuli (*D*_2_*A*_2_), and the residual noise (*H*), which are spatially filtered with *U*.

(3) $$\begin{array}{l} XU=\left(D_{1}A_{1}+D_{2}A_{2}\;+\;H\right)U \end{array}$$

*D*_1_ and *D*_2_ are two real Toeplitz matrices of size *N*_*t*_ × *N*_1_ and *N*_*t*_ × *N*_2_, respectively. *D*_1_ has its first column elements set to zero except for those that correspond to a target onset, which are set to one. For *D*_2_, its first column elements are set to zero except for those that correspond to all stimulus onsets. *A*_1_ and *A*_2_ are two real matrices of size *N*_1_ × *N*_*s*_ and *N*_2_ × *N*_*s*_, respectively. *A*_1_ represents the prototypical ERP in response to targets, and *A*_2_ represents the prototypical ERP in response to all stimuli. *N*_1_ and *N*_2_ are the number of sampling points representing the target and superimposed evoked potentials, respectively. *H* is a real matrix of size *N*_*t*_ × *N*_*s*_.

Let us define spatial filters *U* that maximize the signal to signal plus noise ratio (SSNR):

(4) $$\begin{array}{l} SSNR\left(U\right)=\frac{Tr\left(U^{T}{\hat{A}}_{1}^{T}D_{1}^{T}D_{1}{\hat{A}}_{1}U\right)}{Tr\left(U^{T}X^{T}XU\right)} \end{array}$$

where ${\hat{\text{A}}}_{1}$ corresponds to the least mean square estimation of A_1_:

(5) $$\begin{array}{l} \hat{A}=\left[\begin{array}{c} {\hat{A}}_{1}\\ {\hat{A}}_{2} \end{array}\right]={\left({\left[D_{1};D_{2}\right]}^{T}\left[D_{1};D_{2}\right]\right)}^{-1}{\left[D_{1};D_{2}\right]}^{T}X \end{array}$$

where [D_1_;D_2_] is a matrix of size *N*_*t*_ * (*N*_1_+*N*_2_) obtained by concatenation of *D*_1_ and *D*_2_. Spatial filters are obtained through the Rayleigh quotient by maximizing the SSNR (Rivet et al., 2009). The result of this process provides *N*_*f*_ spatial filters, that are ranked in terms of their SSNR.

Eight spatial filters (*N*_*f*_ = 8) are then used as input to a Bayesian linear discriminant analysis (BLDA) classifier. The input vector is obtained by concatenating the *N*_*f*_ time-course signals across the resulting spatial filters. The BLDA classifier was selected as it is relatively robust to noise in the training data (MacKay, 1992; Hoffmann et al., 2008).

#### Confidence

Confidence measures were derived to identify the reliability of the classification made for each trial. A simple measure, the distance of the classifier score to the discriminating boundary, was used as confidence:

(6) $$Conf=\{\begin{array}{c} \frac{Score-\kappa }{\max\left(Score\right)-\kappa }\;Score>\kappa \\ \frac{Score-\kappa }{\min\left(Score\right)-\kappa }\;Score\leq \kappa \end{array}$$

where *Score* is the score produced by the XD+BLDA classification on a single trial. The classifier score represents a projection from the feature space down to the decision space that maximally separates the two classes. κ is the threshold established through XD+BLDA for discriminating targets from non-target and background distractor stimuli. Max(Score) and min(Score) are the maximum and minimum scores over the entire training set.

#### Performance evaluation

The effect of including the visually-similar non-target stimuli in the RSVP paradigm on classifier performance was explored by comparing the classifier performance across the TO and TN conditions three distinct discriminations. First, target stimuli were discriminated from background distractor stimuli in the TO condition. This discrimination represents the baseline RSVP paradigm with only two types of stimuli. Next, we discriminated target stimuli from background distractor stimuli in the TN condition, omitting the non-target stimuli. Finally, we discriminated target stimuli from both non-target and background distractor stimuli in the TN condition.

For each discrimination, classifier performance was evaluated using a nested 10-fold cross validation with 80% of the data used to train the spatial filter and classifier, 10% of the data used to test the classifier and establish discrimination thresholds, and the remaining 10% of the data used as an independent validation set on which to apply the trained classifier and thresholds. This process was repeated 10 times such that each contiguous 10% slice of data was used as the final validation set. Performance was evaluated based on the area under the ROC curve (Az, Fawcett, 2006) and misclassification rate in the final validation sets.

Misclassification rates were derived based on a discrimination threshold that maximizes the difference between the true positive rate and the false positive rate from the classifier scores in the training set and then applying this threshold to the classifier scores in the validation set. Both Az and misclassification rates were also used to quantify the accuracy of the confidence measures presented here. To do so, a threshold for dividing the data into high confidence and low confidence subsets was varied from 0 to 90% in steps of 10%. A confidence threshold of 0% meant that 0% of the data was included in the low confidence subset, and all of the data was included in the high confidence subset. A confidence threshold of 90% indicated that 90% of the data was included in the low confidence subset and 10% of the data was included in the high confidence subset. For each confidence threshold in this range, the Az and misclassification rates of the high confidence subset were measured. Using these metrics, confidence values that accurately represent the reliability of performance should increase Az and decrease misclassification rates as the confidence threshold is raised.

#### Mitigation strategies

The utility of applying confidence measures was further demonstrated by quantifying the improvement in image labeling accuracy when the estimated confidence was used to trigger a corrective action. This study simulated a simple mitigation strategy where trials above the confidence threshold were classified using the neural classifier and trials below the confidence threshold were manually labeled by the participant. For the purpose of this simulation, we assume a human participant given unlimited time to label the image will attain 100% accuracy, and thus the manually labeled trials were set to the actual image labels. The classification performance using this simulated mitigation strategy was evaluated using Az and misclassification rates for each stimulus class.

## Results

Results across the behavioral, ERP, and single–trial classification analyses demonstrated that adding sparse, visually-similar, non-target images made it more difficult for participants to identity target images.

### Behavior

**Figure 2:**
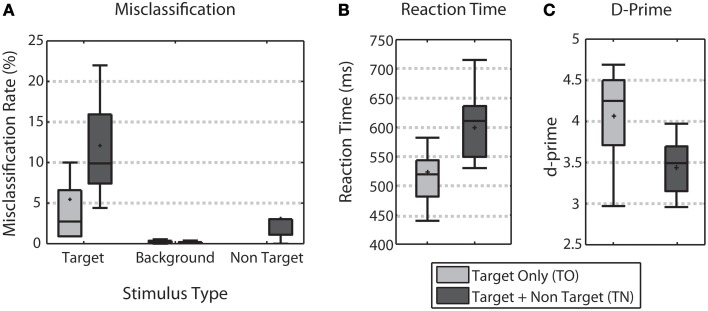
**Behavioral Performance**. **(A)** Shows error rates for each stimulus type for both TO (light gray) and TN (dark gray) conditions. **(B)** Shows target reaction time for both conditions. **(C)** Shows d-prime measures for both conditions. Error bars show the highest and lowest data point within 1.5 times the inter-quartile range of the upper and lower quartiles, respectively. Within each box, crosses indicate mean values and horizontal lines indicate median values.

Behavioral performance was characterized by comparing the error rate by stimulus type, reaction time, and d-prime across the TO and TN conditions (Figure 2). Across all three measures, behavioral performance declined when non-targets were included. Adding non-targets more than doubled the average error rate for target stimuli (difference significant, Wilcoxon signed rank test, *p* < 0.01, Figure 2A). Reaction times obtained from correct target trials were significantly faster in the TO condition (median RT of 514.67 ms) when compared to the TN condition (median RT of 602.82 ms) (Wilcoxon signed rank test, *p* < 0.001, Figure 2B). D-prime analysis showed that target discrimination performance was significantly better for TO trials (median d-prime of 4.25) over TN trials (median d-prime of 3.49) (Wilcoxon signed rank test, *p* < 0.01, Figure 2C).

### ERP analysis

**Figure 3:**
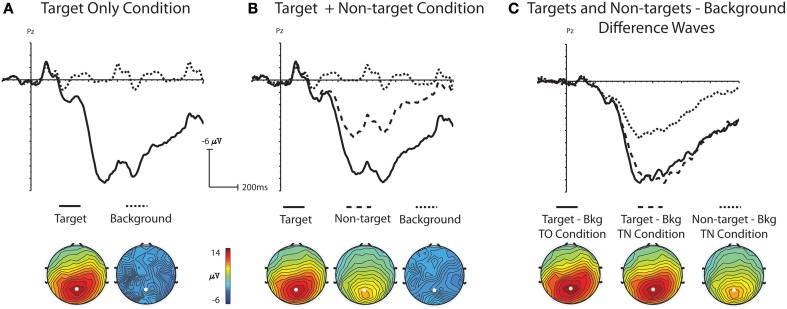
**Grand-average ERP waveforms at electrode Pz and topographic voltage maps (400–800 ms); white dot indicates location of electrode Pz**. **(A)** Shows grand-average ERP waveforms and topographic maps to target and background distractor stimuli in the Target Only (TO) condition. **(B)** Shows grand-average ERP waveforms and topographic maps to target, non-target and background distractor stimuli in the Target plus Non-target (TN) condition. **(C)** Shows difference waves created by subtracting the background distractor from targets in the TO condition and the background distractor from targets and non-targets in the TN condition.

Statistical comparisons of grand average ERP waveforms demonstrated that ERPs were significantly different across stimulus type, with visually-similar non-targets generating ERPs with amplitudes between those of target stimuli and background distracters. In addition, ERPs for background distractor and target stimuli were not significantly different across the TO and TN conditions. A one-way ANOVA was used to analyze the mean amplitude (400–800 ms) from electrode Pz with stimulus (background distractor, target, non-target) as a main factor. There was a main effect for stimulus in the TO condition, [*F*_(1, 16)_ = 111.34, *p* < 0.001], indicating a significantly larger P3 amplitude for targets (mean amplitude: 13.66 μV) relative to background distractors (mean amplitude: −0.44 μV, Figure 3A). A main effect was also obtained in the TN condition [*F*_(2, 32)_ = 83.01, *p* < 0.001]. Subsequent multiple comparison tests using the Tukey-Kramer method showed that amplitudes from background distractors, targets, and non-targets were all significantly different from each other (Figure 3B). A Two-Way ANOVA was run with the factors of Condition (TO or TN) and stimulus (distractor or target) to assess any differences between target P3 amplitude in the two conditions. There was a main effect of stimulus [*F*_(1, 16)_ = 344.33, *p* < 0.001] but no main effect for condition [*F*_(1, 16)_ = 0.001, *p* = 0.978] or interaction [*F*_(1, 16)_ = 0.002, *p* = 0.964] indicating that both the background distractor and target activity was similar between the TO and TN conditions, and that there were significant differences between background distractor and target activity in both the TO and TN conditions (Figure 3).

### Single-trial detection

**Figure 4:**
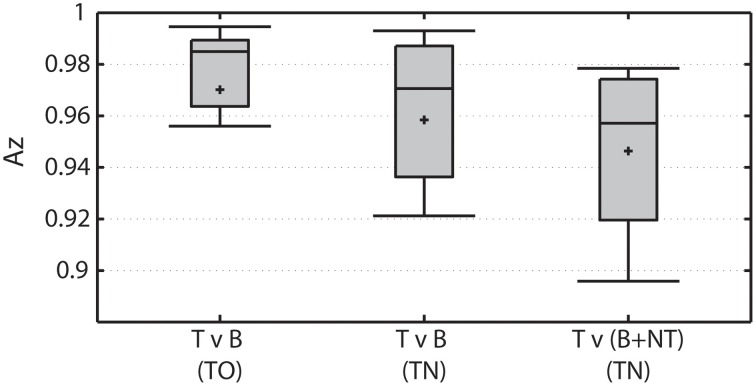
**Overall classification performance under various conditions**. Left: Target vs. background distractor (T v B) discrimination performance in TO condition. Middle: Target vs. background distractor (T v B) discrimination performance in TN condition. Right: Target vs. both background distractor and non-target (T v (B+NT)) discrimination performance in the TN condition.

Overall classification performance declines when visually-similar non-target stimuli are present in the RSVP stream (Figure 4). The TO condition represents the baseline RSVP discrimination of target vs. background distractor. The classifier was highly accurate in this condition, producing average Az > 0.97. When targets are discriminated from background distractor stimuli in the TN condition (ignoring non-target stimuli) performance is not significantly different (Wilcoxon signed rank test; *p* = 0.06). However, when non-target stimuli are included in the discrimination, performance is significantly worse than when they were not included (Wilcoxon signed rank test; *p* < 0.001).

**Figure 5:**
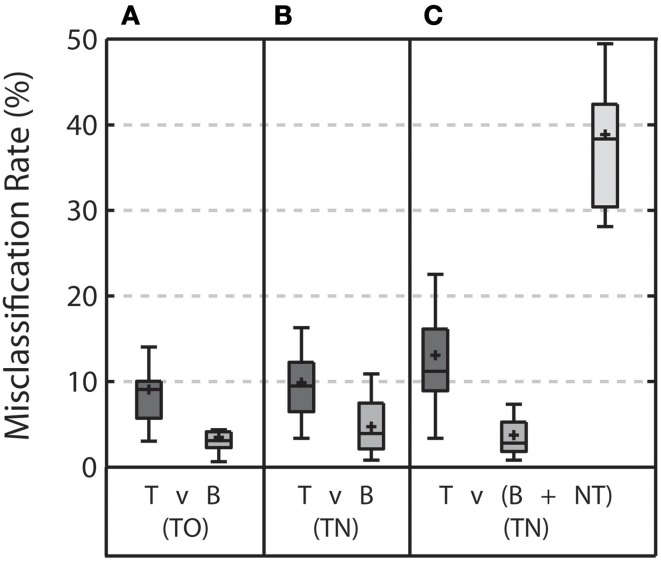
**Misclassification rate for each stimulus type for each discrimination when the threshold was calculated based on the classifier scores from the training set**. **(A)** Shows the misclassification rate for target (T) and background distractor (B) stimuli in the TO condition. **(B)** Shows the misclassification rate for target and background distractor stimuli in the TN condition when targets are discriminated from background distractor only. **(C)** Shows the misclassification rate for target, background distractor, and non-target (NT) stimuli in the TN condition when targets are discriminated from both background distractor and non-target stimuli. Error bars shows the highest and lowest data point within 1.5 times the inter-quartile range of the upper and lower quartiles, respectively. Within each box, crosses indicate mean values and horizontal lines indicate median values.

In addition to the Az measure, the classifier performance was also measured by quantifying the misclassification rate for each stimulus type (Figure 5). Again, we focused on the same three discriminations: target vs. background distractor in the TO condition (Figure 5A), target vs. background distractor in the TN condition (Figure 5B), and target vs. both non-target and background distractor stimuli in the TN condition (Figure 5C). In the baseline TO condition, misclassification rates were below 10% for both target and background distractor stimuli. This level of accuracy would be expected given the high Az levels achieved by in this condition (see Figure 4).

Moving from the TO condition to the TN condition resulted in no significant change in misclassification rates when discriminating target stimuli from background distractor stimuli. (Wilcoxon signed rank test, *p* = 0.23 and *p* = 0.07 for target and background distractor stimuli, respectively). Including non-target stimuli in the discrimination increased misclassification rates for target stimuli (Wilcoxon signed rank test, *p* = 0.01) and resulted in an exceptionally high misclassification rate for non-target stimuli (38.84 ± 8.71%). Misclassification rates for background distractor stimuli were slightly, yet significantly, reduced with the addition of the non-target stimulus (Wilcoxon signed rank test, *p* = 0.049).

**Figure 6:**
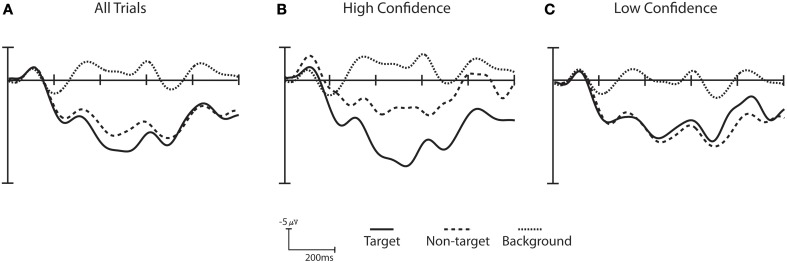
**Confidence ERPs for Subject S10**. **(A)** ERPs across all trials. **(B)** ERPs for the high confidence trials (e.g., top 25% trials when sorted by confidence). **(C)** ERPs for low confidence trial (e.g., bottom 25% trials when sorted by confidence). The difference between the high and low confidence wave form for all three stimulus categories is statistically significant (Wilcoxon signed rank test corrected for multiple comparisons using False Discovery Rate *p* < 0.001). The high confidence trials show a greater separation between target and non-target trials when compared to the low confidence trials.

**Figure 7:**
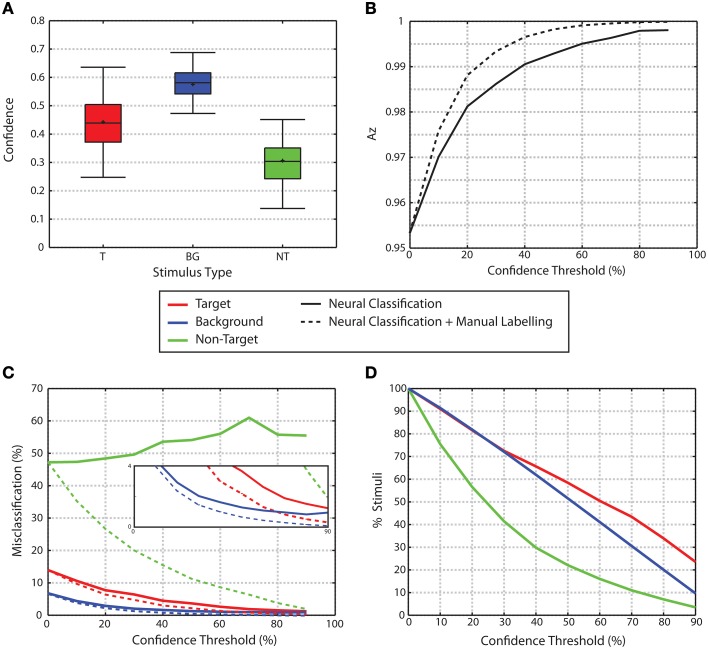
**Confidence. (A)** Confidence levels by stimulus type. **(B)** Az for trials as a function of confidence threshold. Solid line shows the Az for trials exceeding the confidence threshold given. Dashed line shows Az when trials below the confidence threshold are manually labeled while trials above the threshold are labeled through the neural classification. In both cases, as confidence increases, Az increases. **(C)** Misclassification rates for trials that exceed a given confidence threshold. Solid lines show misclassification rates for neural classification only. As confidence increases, the misclassification rates for target and background distractor stimuli fall to nearly 0. Non-target misclassification rates remain high regardless of confidence levels. Dashed lines show misclassification rates when trials below the threshold are manually labeled, while trials above the threshold use neural classification. Misclassification rates for all three stimulus classes are reduced through the manual labeling process. The inset graph show zooms in on the lower portion of the graph to highlight the decrease in misclassification rates for target and background stimuli. **(D)** Percent of trials that exceed a given confidence threshold.

The increase in misclassification rates in the non-target condition is potentially problematic for many real-world applications of this technology where users will encounter instances of non-target stimuli that share the same physical and semantic features as target stimuli. To address this issue, we explored applying confidence measures to the classifier outputs as a means to mitigate the misclassification rate (Figures 6, 7).

Non-target ERPs from high confidence trials are more readily distinguished from target ERPs than in low confidence trials, as shown for subject S10 in Figure 6. Here, high and low confidence trials are defined as the top 25% and bottom 25%, respectively. Trials labeled with high confidence showed greater separation between target trials and both non-targets and background distractor trials than trials with low confidence. A Wilcoxon signed rank test [corrected for multiple comparisons using False Discovery Rate (Benjamini and Hochberg, 1995; Benjamini and Yekutieli, 2001)] shows that the difference between the high and low confidence wave form for all three stimulus categories is statistically significant (*p* < 0.001). When this analysis is extended across all participants, 14 out of 16 participants show significant differences between high and low confidence trials for all three stimulus categories (*p*-values corrected for multiple comparisons using False Discovery Rate, *q* = 0.05). All participants had significant differences between high confidence and low confidence stimuli for at least 2 of the 3 stimulus categories. A similar analysis was carried out to compare behavioral performance between high and low confidence trials (as defined by the classifier), but no significant difference was found.

Overall, non-target stimuli have lower confidence than the target or background distractor stimuli (0.442 ± 0.0057, 0.5751 ± 0.0014, 0.3051 ± 0.0057 mean ± standard error for target, background and non-target stimuli, respectively, Figure 7A). For each participant, a One-Way repeated measures ANOVA was used to analyze the confidence attributed to each stimulus type. When *p*-values are corrected for multiple comparisons using False Discovery Rate analysis (Benjamini and Hochberg, 1995; Benjamini and Yekutieli, 2001), all 16 participants showed a significant effect for stimulus type (*q* < 0.05). Across all participants, the multiple comparisons analysis showed that the confidence attributed to non-target trials was significantly lower than the confidence attributed to both background distractor and target trials for all participants. Additionally, confidence values for target stimuli were less than those for background distractor stimuli.

The use of confidence measures also had a significant effect on classification performance. Figure 7B shows the area under the ROC curve (Az) for classification performance for all trials as a function of confidence thresholds. As the confidence threshold is raised from the minimum to a value that matches 90th percentile of confidence values for each subject, the average Az value across all participants increases to a nearly perfect classification (solid line in Figure 7B). This improvement is further evidenced through the change in misclassification rates for each of the stimulus classes as shown in Figure 7C (solid lines). As the confidence threshold increases, misclassification rates for the target and background distractor stimuli fall to nearly zero. However, non-target stimuli maintain a high level of misclassification regardless of confidence level. The improved performance obtained by raising the confidence threshold comes at the cost of ignoring portions of the data set. The amount of data remaining for each stimulus class for increasing confidence thresholds is shown in Figure 7D. Alternatively however, instead of simply ignoring trials that fall below a confidence threshold, one might instead choose to seek alternative methods for classification. A simple example of an alternative method would be to manually label those images where the neural classifiers failed to produce a highly confident outcome. The performance of such a system improves the overall classification accuracy as shown in the dashed line in Figure 7B at the expense of the extra time needed to manually label images. The performance improvement through the manual labeling process is further evidenced through the reduction of misclassification rates for each stimulus class (Figure 7C, dashed lines). For background and non-target stimuli, the difference between the neural classification alone and the neural classification combined with manual labeling is significant for all confidence thresholds above 0% (Wilcoxon signed rank test *p* < 0.001 for both classes, *p*-values were also corrected for multiple comparisons through False Discovery Rate with *q* < 0.05). For target stimuli, the difference is significant for all confidence thresholds above 0% and < 90% (Wilcoxon signed rank test *p* < 0.001 for both classes, *p*-values were also corrected for multiple comparisons through False Discovery Rate with *q* < 0.05).

## Discussion

Prior work by many groups (Gerson et al., 2006; Bigdely-Shamlo et al., 2008; Parra et al., 2008; Sajda et al., 2010; Touryan et al., 2010, 2011; Cecotti et al., 2011; Yu et al., 2011, 2012; Marathe et al., 2013, 2014b) has demonstrated the effectiveness of using single-trial classification to detect targets in RSVP; however, little of this work explicitly examined how feature similarity between target and non-target stimuli effected target detection accuracy. We addressed this concern in the present study by introducing a more realistic situation where target and non-target stimuli, though each occurred infrequently, shared both physical and semantic features but only targets were task relevant. We evaluated the impact of this manipulation on behavior, ERPs, and single-trial classification of the evoked neural response. Results across the behavioral, ERP, and single–trial classification analyses demonstrated that adding sparse, visually-similar, non-target images made it more difficult for participants to identity target images and more difficult to classify images from neural data.

### Confidence

Previous studies using RSVP-based neural technologies for image triage applications (Gerson et al., 2006; Huang et al., 2008; Mathan et al., 2008; Sajda et al., 2010) have employed statistical methods to identify a subset of trials most likely to be target images. As an extension of this previous work, we employed a confidence-based approach in an offline simulation to mitigate the drop in performance that occurred when non-targets were included in the RSVP stream.

Confidence measures derived from the classifier score were used to sort the data set based on likelihood of correct classification. A comparison of the ERPs and single trial classification performance showed significant differences between the high and low confidence trials. The ERP analysis showed that high confidence target trials were more separate from the non-target and background distractor trials than low confidence target trials. This increased separation led to an improved classification performance for high confidence trials. Specifically, Figure 7B shows that as we remove the lower confidence trials from the performance analysis, classification accuracy improves.

However; the use of a distance from threshold method for establishing confidence, as was done here, has been shown to be less than ideal in previous studies (Platt, 2000). Employing more robust confidence measures (for example, a density-based estimation method in the learned feature space) will likely further improve performance. Additionally, our confidence measures used only information from the classifier scores; however there is potentially a large amount of information in a variety of sources that could further improve the estimate of confidence in a given decision (e.g., data from multiple sensor modalities, individual skill level/expertise, sleep history etc.,). We envision that an accurate estimate of confidence in a particular decision (e.g., target vs. non-target for the current image) may require a combination of a number of the approaches above. Future studies will examine how to improve our confidence estimate by combining different approaches from those listed above. Such endeavors may provide a more robust estimate of confidence that will likely help further improve performance.

Once the low performing trials have been identified, one can employ a number of mitigation strategies. The simplest mitigation strategy would be to simply manually label the low confidence images. If we use the current data to simulate performance when the lowest 20% of trials are manually labeled, overall target detection error is reduced by 36%. While the manually relabeling may be the simplest option, it will dramatically increase the time needed to completely label the set of images. For example, Figure 7C shows that approximately 30% of the data must be manually labeled to reduce the non-target error rate to 20%. If we assume that it takes a user an average of 1 s per manually labeled image, then the manual labeling will increase the total labeling time by 60%. While this increased labeling time may be acceptable for some applications, other strategies may be more efficient. For example, the low confidence images can be re-displayed to the same person using RSVP, or sent to another person for target identification. Alternatively, we may also be able to couple the human based target identification with an automatic target recognition system (Wang et al., 2009; Sajda et al., 2010) to improve performance. Such an endeavor is currently underway (Marathe et al., 2014a) and will greatly benefit from the results presented here.

The improvement demonstrated by the inclusion of confidence measures has broad implications for the development of future systems. While we focused on an RSVP-based target detection paradigm, the use of confidence in human decisions can be extended to a wide range of human-in-the loop systems. The principle of confidence has been applied in control theory to account for variable or noisy sensors. Here we provide initial evidence that the same principle can be applied to account for inherent variability in human decisions.

### Top-down influences

One aspect that was not explored in this study was how top-down influences due to task instructions may have affected performance. In this study participants were told to explicitly look for people with weapons in order to test whether the participants and subsequently the classification algorithms could discern people with weapons (targets) from people without weapons (non-targets). The ERP analysis suggests that early stages (200–400 ms) of the P3 waveform may reflect an orienting response to stimulus novelty since rare target and non-target waveforms were similar but different from the frequent background distractors. Later stages (400–600 ms) of the P3 show differences between targets, non-targets and background distractors indicating processes related to target selection or non-target inhibition. Since both targets and non-targets shared many properties (appearing infrequently, people) participants may have adopted a strategy to orient to any rare stimulus. Other research that included a non-target stimulus in a standard oddball paradigm showed that non-targets have a neural response similar to the frequent background distractors and not the target (Steiner et al., 2013); however the stimuli used in this study were simple shape stimuli containing different stimulus properties, e.g., circles, squares, triangles. This may have lead participants to select targets or possibly inhibit non-targets at an earlier stage of processing than what was seen in the current study. The ERP waveforms and classification results may have been different if participants searched for targets that did not contain features similar to non-targets (Polich and Comerchero, 2003), or if the instructions had been to explicitly look for weapons (with no mention of people).

## Conclusion

By evaluating the impact of adding a non-target stimulus to a standard RSVP-based paradigm, this study begins the process of moving RSVP based target identification applications into more complex environments that include natural images. We have shown that the introduction of a non-target stimulus yields a significant slowing of reaction time and reduction of d-prime. This decrement in behavioral performance is accompanied by a decrement in classification accuracy for single-trial detection and an increase in misclassification rates. Importantly we show that incorporating measures of confidence can identify trials where the drop in performance is likely to occur. Using confidence measures, we enable these systems to employ a number of possible mitigation strategies that enable the integration of information from alternative sources as a means to improve classification performance.

### Conflict of interest statement

The authors declare that the research was conducted in the absence of any commercial or financial relationships that could be construed as a potential conflict of interest.
